# PrEP Stigma as a Minority Stressor among Black Sexual Minority Men: A Mixed-Methods Study

**DOI:** 10.1007/s10461-024-04481-1

**Published:** 2024-09-17

**Authors:** Rodman Turpin, Aaron D. Camp, C. J. Mandell, Julia Mandeville, Rochelle R. Davidson Mhonde, Jamil Smith, Hongjie Liu, Typhanye Dyer, Kenneth H. Mayer, Bradley Boekeloo

**Affiliations:** 1 Department of Global and Community Health, College of Public Health, George Mason University, Fairfax, VA, USA; 2 Heller School for Social Policy and Management, Brandeis University, Waltham, MA 02453, USA; 3 INOVA Health System, Fairfax, VA 22031, USA; 4 Grady Health System, Atlanta, GA 30344, USA; 5 Department of Epidemiology and Biostatistics, School of Public Health, University of Maryland, College Park, MD, USA; 6 The Fenway Institute, Fenway Health, Boston, MA, USA; 7 Department of Medicine, Beth Israel Deaconess Medical Center, Harvard Medical School, Boston, USA; 8 Department of Behavioral and Community Health, School of Public Health, University of Maryland, College Park, MD, USA

**Keywords:** HIV, LGBT, Prevention, Stigma, Mixed methods

## Abstract

Black sexual minority men (BSMM) remain disproportionately affected by HIV, yet Pre-exposure prophylaxis (PrEP) uptake in this population remains relatively low. Informed by minority stress theory, PrEP stigma may manifest in and exacerbate societal marginalization based on sexuality and race. We used an exploratory sequential mixed-methods approach to determine if PrEP-specific stigma was associated with reduced PrEP uptake among BSMM, and qualitatively explored how PrEP use is stigmatized among BSMM. We analyzed cross-sectional data from a pilot sample of BSMM (*n* = 151) collected in late 2020 in the United States, testing for associations between PrEP stigma and PrEP use using modified Poisson regression. Subsequently, we selected participants (*n* = 23) from this sample for qualitative interviews starting in 2022. Responses to questions related to PrEP stigma were analyzed using thematic analysis. PrEP stigma was associated less than half the PrEP use (aPR = 0.43, 95% CI = 0.24, 0.75) among BSMM after adjustment. Qualitatively, we identified three major themes in how PrEP use is stigmatized among BSMM: PrEP-specific sexual stigma, intersections between PrEP and HIV stigma, and PrEP misinformation and disinformation. Aligned with minority stress theory, each theme was based in part in stigma related to sexuality or race. We found strong relationships between PrEP stigma and PrEP use independent of several sociobehavioral factors. Each of our themes were based in part in minority stressors, and underscore the importance of culturally competent PrEP promotion efforts towards BSMM. Addressing stigma is a core component of health equity efforts towards ending the HIV epidemic.

## Introduction

Black sexual minority men (BSMM) are disproportionately affected by HIV, accounting for approximately 26% of all new diagnoses in the United States (U.S.) despite comprising less than 1% of the population [1]. Among BSMM aged 25 to 34, HIV diagnoses have continued to increase since 2010 [1, 2]. It is projected that, if current trends continue, one in two BSMM will be diagnosed with HIV in their lifetime [3]. PrEP has been shown to significantly reduce HIV incidence across various populations, including sexual minority men (SMM), and is a key component in HIV-prevention efforts [4, 5]. This is especially true for populations more vulnerable to HIV, such as BSMM [7]. However, uptake of PrEP has been lower among BSMM compared to White or Hispanic/Latino SMM, despite their higher HIV incidence rates [6, 7]. For these reasons, public health efforts are increasingly focused on understanding and addressing barriers to PrEP use among BSMM [8–10].

Minority stress theory suggests that adverse health outcomes are linked to societal marginalization based on identity-related characteristics, such as sexuality and race [11]. These outcomes may include depression, suicidality, substance use, and adverse health behaviors [12–14]. This theory has been applied to understand the health outcomes of several LGBTQ + populations, including BSMM [15, 16]. Extant research indicates that stigma and discrimination, based on both sexuality and race, contribute to adverse health outcomes in BSMM, including substance use, condomless sex, and negative HIV-related outcomes [17, 18]. This marginalization may also deter BSMM from adopting HIV-prevention measures like PrEP uptake, which is an important contributor to HIV disparities, with far-reaching implications for public health efforts to combat the HIV epidemic [19].

In many ways, stigmatizing and negative attitudes towards PrEP use may be a form of minority stress, stress directly related to minoritized identity that results in adverse health outcomes [11]. First, perceptions of PrEP users as sexually “high risk”, while partially informed by targeted PrEP efforts towards those with greater HIV vulnerability, is associated with sexual stigma that may be a substantial barrier to PrEP use [20]. This has been documented in some literature, though nuances on how PrEP-specific stigma operates among BSMM (e.g., internalized vs. experienced vs. anticipated), and what forms are the most prevalent need further study [21]. Understanding these nuances are critical for effective stigma-reduction interventions to promote PrEP use in this population. Second BSMM face multiple minoritized experiences, including stigma related to both sexuality and race, consistent with intersectionality theory as well [22]. Given the greater resistance to PrEP uptake among BSMM compared to other racial/ethnic groups of SMM [7], and that different stigmas often mutually reinforce one another (i.e., one form of stigma may lead to or exacerbate another form of stigma), stigma related to race may also manifest in negative PrEP attitudes [22]. Exploring how PrEP stigma is experienced and communicated by BSMM may further elucidate how it functions as a minority stressor, with important implications for PrEP prevention efforts.

Our study used an explanatory sequential mixed-methods approach to answer our research questions: Is PrEP-specific stigma associated with reduced PrEP uptake among BSMM, and how is PrEP use stigmatized among and towards BSMM? Based on minority stress theory, we hypothesized that PrEP stigma would be largely based in minority-related stressors, including racial and sexual minority discrimination, violence, sexual stigma, and other factors related to sexuality and race.

## Methods

### Sampling and Integration

We conducted an explanatory sequential mixed-methods with two data sources, using both connecting (i.e., informing qualitative sampling based on our quantitative study) and building (i.e., informing qualitative measurement based on our quantitative study) approaches to integrate our studies [23]. Initially, we examined cross-sectional data from a group of 151 HIV-negative BSMM in the U.S. collected in late 2020 [24]. Recruitment occurred through BSMM-specific social media platforms like Jack’d and Grindr, as well as BSMM community-serving organizations primarily in the D.C. Metropolitan area. Eligibility criteria included: Being age 18 years or older, identifying as Black, African American, African, or Afro-Caribbean, identifying as male, having a male sexual partner in the last 6 months, and being HIV-negative. The University of Maryland Institutional Review Board (IRB) granted approval for the study, and all participants provided written informed consent. Using a connecting approach, we selected 23 participants from this larger sample for qualitative interviews starting in 2022. The subsample was selected to approximate the original sociodemographics of the quantitative sample, through initial random selection for the first 18 participants, and more purposive selection for the last 5. Purposive selection aimed to maintain the socioeconomic and age diversity of our original sample, as PrEP stigma was present across all sociodemographic levels. Those expressing interest and meeting eligibility criteria underwent in-depth interviews within the next week, reviewing an electronic consent form in advance. Next, we utilized a building approach to inform measurement of PrEP stigma. The questions asked were related to many of the items in the original PrEP stigma measurement (e.g., quantitative questions related to community-based PrEP stigma informed the use of questions such as “Do you think PrEP is accepted in the Black queer community?” in the qualitative interviews”). The George Mason University and University of Maryland IRB approved all qualitative study activities. Findings from the qualitative component were interpreted within the context of the quantitative associations (e.g., if PrEP stigma is strongly associated with lower PrEP use, each qualitative PrEP stigma theme is interpreted in how it ultimately may lead to lower PrEP use).

### Procedures

#### Quantitative Survey Procedures

Our primary exposure was PrEP stigma, measured using the 10-item PrEP Stigma and Positive Attitudes scale [21]. This scale consists of items reflecting stigmatizing beliefs regarding PrEP and PrEP users (e.g., “People who are on PrEP are irresponsible”), with higher values reflecting greater stigma. This scale demonstrated strong internal consistency in our sample (Cronbach’s alpha = 0.85). Current PrEP use (yes/no) was our outcome. Covariates selected based on prior associations in the literature with both stigma and PrEP use [21, 25], included age, highest education level, region, sexual identity, relationship status, health insurance, sexual partner concurrence (i.e., if any of your sexual partners have any other sexual partners), and depression measured using the PHQ-9 [26].

#### Qualitative Interview Procedures

In-depth interviews were conducted via phone among our qualitative sample. The interviewer also discussed the consent form in detail at the start of the interview, answered any questions, and received verbal informed consent from all participants. Interviews used a semi-structured interview guide and took 15–25 min each. Interview questions were directly informed by the quantitative study, particularly our findings regarding relationships between PrEP stigma and PrEP use, as we sought to better understand this relationship. Questions for this study were focused on sociodemographics (e.g., age, sexual identity) and PrEP stigma (e.g., “Do you think PrEP is accepted in the Black queer community?”). All interviews were conducted by the study team lead, who is a member of the BSMM community and experienced in BSMM community-based health service. Participants were compensated $30 for each interview.

#### Qualitative Data Management

All interviews were recorded in audio format and transcribed using a two-step process. Initially, interviews were transcribed using Descript, an automated transcription service, which converted them into editable text suitable for further processing [27]. Subsequently, three members of the analysis team reviewed both transcripts and audio, rectifying any errors in the initial automated transcription. Given that the initial automated transcription was approximately 95% accurate, this method proved efficient. The transcript data was then managed using Descript and Microsoft Word. Various precautions were implemented to safeguard data security and confidentiality. These precautions included analyzing audio data exclusively on encrypted, password-protected computers disconnected from public networks, ensuring audio data never left the premises, deleting audio data from Descript after transcription, and eliminating any identifiable information (e.g., names) from transcripts.

### Analyses

#### Quantitative

Data was collected using online surveys. We tested associations between PrEP stigma and our binary PrEP use measure using modified Poisson regression with robust standard errors [28]. This was a useful method for generating prevalence ratios that allowed for inclusion of more confounders than log-binomial modeling. We generated unadjusted models and models adjusted for age, highest education level, sexual identity, relationship status, health insurance, sexual partner concurrence, and depression. Region was not included due to excessive covariance with other sociodemographic covariates. For all models we generated ratio estimates and 95% confidence intervals. Missingness for all items was low (less than 10%), with most items having less than 2% missingness. We used intrascale stochastic imputation to impute missing items from other items within both the PrEP stigma and depression scales. The overall low missingness and strong internal consistency of each scale supported this approach. We retained all observations post-imputation (*n* = 151). All bivariate and regression analyses were conducted in SAS 9.4 [29].

#### Qualitative

A team consisting of two faculty members, two graduate students, and one additional research colleague analyzed all interview data. An inductive approach was guided by thesix phases of thematic analysis, with subsequent assessment of how themes aligned with minority stress theory [30]. Initially, a team member individually read and reread each transcript, noting topics of interest and questions (phase 1 – becoming familiar with the data). Subsequently, passages were identified, and common themes were described (phase 2 – generating initial codes). The analysis team met biweekly to review identified passages and codes. The primary coder then independently reviewed interviews for common themes (phase 3 – searching for themes) and categorized text passages based on these themes. A secondary coder performed an interrater reliability check, assessing agreement between the two reviews and all primary codes. Any discrepancies were resolved in meetings. After primary and secondary coding, the primary coder reviewed all coded passages, generating thematic keywords and phrases that specifically described the main topics (phase 4 – interpreting the themes). The analysis team collectively identified common keywords as main themes and less common ones as sub-themes (phase 5 – refining the specifics of the themes). The final list of themes was inductively interpreted by the entire research team (phase 6 – final analysis), and then assessed for consistency with minority stress theory.

## Results

### Sample and Bivariate Analyses

Approximately half of the sample was between the ages of 25 and 34 (Table 1), 84.8% had health insurance, and 61.6% had completed an undergraduate or graduate college degree. Just over half (57.0%) identified as gay, with 19.9% identifying as bisexual. Nearly half were single (44.4%) and 43.1% reported sexual partner concurrence. The median PHQ-9 score was 15, this cutoff indicating moderately severe depression. Just over a quarter (28.5%) of participants reported currently using PrEP. Greater PrEP stigma was associated with lower education, lack of health insurance, greater depression, and not using PrEP. Additionally, lower education, and heterosexual and bisexual identity were also associated with lower likelihood of using PrEP.

### Regression Analyses

PrEP stigma was associated with lower odds of currently using PrEP in all models. Compared to unadjusted models (PR = 0.74, 95% CI = 0.61, 0.90), adjusted models demonstrated noticeably stronger estimates, with just over double the proportion of not using PrEP per 50-unit increase in PrEP stigma. This unit range reflects the approximate interquartile range of PrEP stigma in our sample. Notably, age accounted for nearly all of the detected confounding, as age-adjusted estimates (aPR = 0.47, 95% CI = 0.31, 0.74) were very similar to fully adjusted estimates (aPR = 0.43, 95% CI = 0.24, 0.75). Among covariates, only monogamous relationships were associated with lower PrEP use, though sexual partner concurrence was not. Age group 25–34 was marginally (0.05 < *p* < 0.10) associated with lower PrEP use (aPR = 0.63, 95% CI = 0.34, 1.17) (See Table 2).

### Qualitative Sample Characteristics and Themes

The qualitative subsample was similar to the quantitative sample across all measures (Appendix 1), with the exception of moderate differences in proportions age 35 or older (13.1% proportion difference) and gay sexual identity (19.8% proportion difference). Saturation of themes was achieved in 18 interviews. We identified three major themes from our thematic analysis: PrEP-specific Sexual Stigma, Intersections between PrEP and HIV Stigma, and PrEP Misinformation (Table 3). Participant responses are provided in italics, with pseudonyms and age ranges (in parentheses) for all participants in quotations.

### PrEP-specific Sexual Stigma

The most commonly described theme was PrEP being stigmatized directly related to sex and sexuality (91.3%). The conflation of PrEP and hypersexuality, or stigmatized forms of sex (e.g., receptive anal intercourse, casual sex, having more than one sexual partner) was a driver of PrEP stigma reported by almost every single participant in our qualitative sample. This is summarized succinctly by one participant, “Bryce (25–34)”, when asked to elaborate on how people who use PrEP are stigmatized:

Sex is usually like the biggest one…what type of sex you have or wanna have, how you engage with, who you engage with, how many people you engage with.

Participants described internalized, experienced, and anticipated sexual stigma specific to PrEP use. While some participants noted that PrEP use was steadily increasing among BSMM, internalization of PrEP stigma among the population was still a substantial issue. “Derrick (18–24)” demonstrated some of this internalized stigma when asked why he wouldn’t consider using PrEP:

It’s a certain kind of gay (person), a promiscuous gay (person), is who (PrEP) is for. I wouldn’t say they’re nasty, but they’re not clean either.

Internalization of negative PrEP attitudes directly stemmed from conflation of PrEP with stigmatized forms of queer sex, and the devaluing of sexual minority people who engage in these forms of sex. In this way, PrEP stigma is a form of internalized sexual minority stigma. “Hakeem (25–34)” described how moral judgment towards casual sex translates into judgment towards PrEP use:

People who might not like the idea of casual or anonymous sex might wonder why PrEP is being given to some of these…what’s the word I’m looking for… sluts, perhaps. I could probably find a better word, but sluts is what came to mind. And yeah, I think there could definitely be some moral grandstanding, particularly that viewpoint in regard to PrEP use.

Even for participants where internalization of negative PrEP attitudes, both experienced and anticipated PrEP stigma were described as barriers to PrEP use. Notably, every participant who reported some form of experienced sexual stigma related to PrEP also reported anticipated sexual PrEP stigma. “Hakeem (25–34)” further describes this when asked to elaborate on his experiences related to sexual stigma, and how it relates to PrEP use:

Having unprotected sex, you know, makes you dirty…. people treat you like you’re not deserving of love. You’re not deserving of success. You’re not deserving of just being able to live a normal life.

This form of PrEP stigma relied on both assumptions regarding the kind of sex that BSMM on PrEP were engaging in (i.e., condomless sex), as well as stigmatization of those forms of sex. The anticipation of stigma was impactful enough to deter some BSMM from engaging with PrEP services at all, to avoid stigmatizing assumptions regarding sex and sexuality. “Najee” (35–50) discussed this in reference to PrEP services:

Um, but at the same time, another stigma that I have experienced as well is people shy away from (PrEP) services and conversations about those services because they don’t want their tea possibly being spilled. They’d rather not visit those places or even talk about those places just to keep a certain image to their peers.

Here, the “tea possibly being spilled” refers to vulnerable secrets, in this case stigmatized sexual activity. Participants reported not wanting to have assumptions made regarding either their sexuality, or their sexual activity, which resulted in distancing and negative attitudes towards PrEP use. “Kelvin” (18–24) concisely demonstrates this when asked why he would not consider seeking PrEP services:

If I asked you for PrEP, you would assume I’m busting it open for everyone. Right?

### Intersections between PrEP and HIV Stigma

The second identified theme was HIV Stigma as a driver of PrEP stigma. This was described as closely related in part because PrEP is related to HIV as a form of prevention, but also shares various stigmas with HIV, particularly related to sex. “Roy” (25–34) mentions this:

I mean, stigma intersects, when we’re talking about HIV, being positive, or getting on PrEP. Both of those things are stigmatized.

A notable link between HIV stigma and PrEP stigma participants noted was related to accessing PrEP services, specifically concerns that people would assume they have HIV because they are getting services at venues that provide HIV care; some participants noted that community-based venues for PrEP also focus on HIV care linkage and services as well. “Quan” (35–49) describes this as related to perceptions in the BSMM community:

If you are not out, just being seen there, people can connect those dots… just going into those spaces that deal with the HIV community (is difficult), and if you’re seen at those (spaces), then it’s like, “oh my gosh, do they have HIV as well?” But it’s like, “no, I’m just there to get PrEP.

Here in addition to the concerns about being outed when seen at BSMM-serving organizations, the assumption that one is going for HIV care services was described by multiple participants as a source of negative attitudes towards PrEP. The fear of peers assuming an individual is HIV positive was distinctly noted as an intracommunity concern. Conversely, an externally-driven source of PrEP stigma among BSMM was based on assumptions about HIV due to race and sexuality. Some participants noted that many BSMM are exhausted with assumptions that they have HIV, and the associated stigma related to HIV and HIV risk. “Bryce (25–34)” briefly describes this:

Because a lot of people think that Black gay and bisexual men are, quote, “sick” unquote. “And it turns us (BSMM) off of prevention, like PrEP.

Here, “sick” is a stigmatizing term used to refer to people living with HIV. Participants noted that stigmatizing views regarding people living with HIV were directly conflated with being a Black sexual minority man. This was especially true regarding assumptions of “risk” based on being Black and queer. This resulted in more negative attitudes towards PrEP as a rejection of that conflation. “Lucius (25–34)” described his resistance to PrEP as deeply rooted in how his Black, gay identity is assumed to be inherently “high-risk” for HIV:

I just, I don’t really understand why everything has to be HIV when you’re Black and gay. We don’t all have HIV. We shade each other for that too much. So, I’m not interested in another thing (i.e., PrEP) that marks, that gives me that high-risk (for HIV) label simply because I happen to be Black and gay.

“Shade” here refers to intracommunity stigma, in this case BSMM stigmatizing each other based on having, or assumptions of having HIV. This highlights how this type of stigma manifests in intracommunity interactions, and both present barriers to PrEP engagement.

### PrEP Misinformation and Disinformation

Finally, PrEP misinformation and disinformation emerged as our last theme. Many participants described an overall lack of knowledge as a persistent barrier to PrEP use. “Jabari” (25–34), a PrEP ambassador for several years, describes how common these issues are in PrEP promotion:

(Stigma) affects PrEP as far as people being ignorant. I’ve been a PrEP ambassador here (in D.C.), in Texas, and in Illinois. And a lot of the dialogue that I’ve had with people was dialogue out of ignorance. You know, it was just a lot of things that they did not know, and they were like privy to this preconceived notion of things that they have heard in their friend groups.

There were two ways in which misinformation related to PrEP was discussed as a source of negative PrEP attitudes. One was an overestimation of the harms or difficulties related to using PrEP. “Nick” (35–49) discusses a false conflation between PrEP regimens and HIV care regimens when discussing PrEP with a friend who was resistant to considering it:

So (he said) I don’t want to take all those pills every day. And you know, then I had to redirect it by saying, no, no, this is 1 pill a day….Again, it’s because of lack of knowledge….because for (some HIV) treatments, it requires you taking a daily cocktail.

Here, misinformation about how PrEP is taken created a barrier to even the consideration of taking the preventative medication. Much of the misinformation around PrEP was related to how it is consumed, efficacy, and side effects. “Lucius” (25–34) described his hesitancy towards PrEP use based on misinformation related to both PrEP efficacy and PrEP side effects:

But does it prevent HIV? I don’t think we can say that for sure. Now we do know it can make you sterile…. this is what (the medical system does) to us. And I’m not even pressed for kids, but if it can do that then what else (can) it do?

When asked to elaborate on where he heard this information, he said this originated from an article shared on social media. Multiple participants noted that social media was a source of misinformation and disinformation on PrEP use. The context of medical mistrust here (i.e., “this is what the medical system does to us”) is referring to historical and current medical abuses of Black people. This deep-rooted mistrust facilitates greater vulnerability to medical misinformation. In totality, this highlights the role of accurate and culturally competent health information in destigmatizing PrEP among BSMM, especially in the context of minority stressors related to race.

## Discussion

Using a mixed-methods approach, we identified associations between PrEP stigma and PrEP use, and found PrEP-specific sexual stigma, intersections between PrEP and HIV stigma, and PrEP misinformation and disinformation as major themes in how PrEP use is stigmatized. Our quantitative findings, while intuitive, also have some important nuances. After age-adjustment, PrEP stigma was associated with more than a two-fold decrease in PrEP use, independent of any additional covariates other than age. This stigma is critical to address in PrEP promotion efforts, consistent with much of the literature on the negative effects of stigma [18, 19]. In fully adjusted models, being in a monogamous relationship was associated with lower PrEP use, but sexual partner concurrence was not. These both capture a similar form of sexual network risk (e.g., if your partner has other partners), and both are being perceived by the participant. Thus, monogamous relationship status may deter PrEP use through mechanisms other than perceived sexual risk, such as relationship expectations and agency; this warrants future study. Additionally, bisexual and heterosexual-identifying participants had the lowest rates of PrEP use despite having male sexual partners (as this was part of the inclusion criteria). These differences may underscore differences in minority stress based on sexual identity, particularly related to opposite gender relationships among men who have sex with men and women and the pressure to conceal sexual identity to avoid stigma [30]. This is heavily rooted in sexual stigma characterized by pressure to avoid PrEP service providers for fear of “being outed”. Participants described this sexual stigma as the most common way PrEP is stigmatized, a finding that has been well-demonstrated in the literature [19–21]. Additionally, the stigma towards forms of sex that sexual minority people engage in was a direct barrier to PrEP use; this aligns with minority stress theory [11, 12]. Destigmatization of sexuality is especially important to PrEP promotion in sexual minority communities, including BSMM, as internalized sexual stigma can compromise access to HIV prevention services [20].

The relationships between HIV stigma and PrEP stigma have significant relevance to PrEP promotion efforts [19]. In some ways, this appears counterintuitive, as PrEP prevents HIV. They are both largely drawn from similar negative attitudes towards sexuality, however. Within this theme were two important findings. First, consistent with minority stress theory, much of the HIV stigma was described as rooted in race and sexuality. This is aligned with much of the literature on Black queer experiences related to stigma, including studies based in intersectionality theory [18, 23]. Second, HIV is still so stigmatized within BSMM communities that the appearance of possibly having HIV is enough to deter engagement with prevention. Taken in tandem, this highlights how intracommunity presentation, particularly related to sexual stigmatization, is a critical factor in BSMM decision to seek PrEP services. This presents challenges in delivering care services, particularly in reaching parts of the BSMM community that are especially stigmatized. The utilization of social venues (e.g., bars, clubs) may circumvent some of these limitations, as these are non-clinical spaces that do not have inherent associations with HIV care or prevention. While this is a potential “workaround”, the deeper issue of how sexuality and HIV are stigmatized is critical to address, both in intracommunity and intercommunity contexts. Participant discussions about intracommunity stigma (i.e., “shade”) were especially salient in this sample. Stigma towards HIV and sexuality both are associated with substantial harms among sexual minority people [18–20, 22, 31]. This is especially true for BSMM regarding HIV stigma, as the increased vulnerability to HIV also creates increased vulnerability to HIV stigma. Addressing these forms of stigma is necessary for optimal delivery of PrEP services to this community, with important implications for combating the HIV epidemic at large [1].

Finally, misinformation and disinformation regarding PrEP is a well-documented barrier to PrEP utilization [32]. Disinformation in particular is prevalent on social media, which multiple participants noted as a source of incorrect views regarding PrEP [32]. Among BSMM participants, this was rooted in prominent medical harms towards Black communities, both historical and current. In this way, the vulnerability to misinformation and disinformation is rooted in minority stress related to race. In totality, each of these themes were based in part on minority stressors, and underscore the importance of culturally competent PrEP promotion efforts towards BSMM.

There are important strengths to our research to consider. We were able to identify major themes that clarified how PrEP stigma manifests among BSMM, built upon identified quantitative relationships between PrEP Stigma and lower PrEP Use. The mixed-methods approach provides important context to the initially identified quantitative relationships, particularly by achieving thematic saturation and gaining an in depth understanding of participant experiences. A strong rapport was established with participants, partly because the research lead (who is also the interviewer) is a member of the BSMM community and well-connected to BSMM community-serving organizations. Finally, this study fills a gap in the existing literature by exploring nuanced aspects of PrEP stigma in this population. This study has limitations as well. As our study focuses on BSMM, the findings might not reflect the experiences of SMM from other racial/ethnic groups. However, focusing on PrEP among BSMM is scientifically justified due to their heightened vulnerability to HIV. Additionally, the sensitive nature of many topics may be influenced by social desirability bias. Despite these limitations, participants shared numerous vulnerable and stigmatized experiences, partly due to the trusting rapport established with the interviewer.

## Conclusion

Using an explanatory sequential mixed-methods approach, we found quantitative relationships between PrEP stigma and PrEP use independent of several sociobehavioral factors, and qualitatively identified three major themes in how PrEP use is stigmatized: PrEP-specific sexual stigma, intersections between PrEP and HIV stigma, and PrEP misinformation and disinformation. Aligned with minority stress theory, each of these themes were based in part on stigma related to sex, sexuality, or race. Cultural competence towards BSMM communities, including both internal and external stigma reduction, are of great importance in PrEP promotion efforts. Future research exploring intracommunity barriers to PrEP use among BSMM, particularly related to social presentation and relationship contexts, is recommended, as is research focused on age-differential barriers to PrEP use in this community. Addressing stigma is necessary to effective HIV-prevention efforts for BSMM, and thus are a core component of health equity efforts towards ending the HIV epidemic.

## Figures and Tables

**Table 1:** Sample characteristics, median PrEP stigma, and PrEP use among BSMM (*n*=151)

	Total (%)	PrEP Stigma		PrEP Use	
		(Median)	Statistic	(%)	Statistic
**Age group** ^1^					
18–24	12.7	50.0	ρ = −0.07	33.3	Z = 0.34
25–34	48.1	30.0		26.2	
35–49	39.2	40.0		28.6	
**Highest education level** ^1^					
High school or less	13.9	**45.0**	ρ = −0.18*	**23.8**	Z = −0.43
Some college	24.5	**48.8**		**24.1**	
College (undergraduate degree)	35.8	**46.3**		**32.4**	
College (graduate degree)	25.8	**40.0**		**33.3**	
**Region** ^2^					
Northeast	59.5	35.0	χ^2^ = 1.31	*31.4*	χ^2^ = 3.08
West/Midwest	9.8	47.5		*11.7*	
South	31.7	40.0		*29.8*	
**Sexual identity** ^2^					
Bisexual	20.3	42.5	χ^2^ = 1.11	**20.0**	χ^2^ = 4.42*
Gay	63.3	35.0		**30.2**	
Heterosexual	3.8	45.0		**17.7**	
Blaqueer/SGL/Queer	12.7	41.3		**44.4**	
**Relationship status** ^2^					
Single	44.3	45.0	χ^2^ = 2.68**	**34.3**	χ^2^ = 6.72*
Dating	17.7	36.3		**34.6**	
Partnered—monogamous	31.7	38.8		**13.6**	
Partnered—non-monogamous	6.3	35.0		**35.7**	
**Current health insurance** ^2^					
Yes	84.8	**36.3**	Z = −3.33**	*31.2*	χ^2^ = 2.22
No	15.2	**50.0**		*17.2*	
**Sexual partner concurrence** ^3^					
Yes	56.9	40.0	Z = 0.15	31.1	χ^2^ = 0.82
No	43.1	40.0		27.1	
**Depression above median** ^3^					
Yes	56.3	**47.5**	Z = −2.75**	28.0	χ^2^ = 0.01
No	43.7	**35.0**		28.6	
**Current PrEP use** ^3^					
Yes	28.5	**27.5**	Z = 2.74**	-	
No	71.5	**45.0**		-	

1 PrEP Stigma differences tested using Spearman’s rank-sum test. PrEP use differences tested using Cochran-Armitage test of trend

2 PrEP Stigma differences tested using Kruskal-Wallis test. PrEP use differences tested using binarized Chi-Square test

3 PrEP Stigma differences tested using Cochran-Armitage test of trend. PrEP use differences tested using Chi-Square test

* P < 0.05.

** p < 01

Estimates where *p* < 0.005 are bolded. Estimates where 0.05< *p* <0.10 are italicized

**Table 2:** Unadjusted, age-adjusted, and fully adjusted associations between PrEP stigma and currently using PrEP among BSMM and 95% confidence intervals (*n*=151)

	Unadjusted	Age-Adjusted	Fully Adjusted
**PrEP stigma** ^1^	**0.74 (0.61, 0.90)** **	**0.47 (0.31. 0.74)** **	**0.43 (0.24, 0.75)** **
**Age group**			
18–24		Reference	Reference
25–34		*0.66 (0.37*, *1.18)*	*0.63 (0.34*, *1.17)*
35–49		0.78 (0.43, 1.39)	0.92 (0.47, 1.79)
**Highest education level**			
High school or less			Reference
Some college			1.30 (0.64, 2.63)
College (undergraduate degree)			0.97 (0.50, 1.88)
College (graduate degree)			1.25 (0.59, 2.68)
**Sexual identity**			
Bisexual			0.70 (0.40, 1.24)
Gay			Reference
Heterosexual			0.84 (0.37, 1.90)
Blaqueer/SGL/Queer			1.56 (0.77, 3.19)
**Relationship status**			
Single			Reference
Dating			1.09 (0.55, 2.17)
Partnered—monogamous			**0.39 (0.20, 0.78)** **
Partnered—non-monogamous			0.84 (0.37, 1.92)
**Current health insurance**			1.50 (0.79, 2.85)
**Sexual partner concurrence**			0.97 (0.48, 1.96)
**Depression above median**			1.00 (0.97, 1.03)

* *p* < 0.05.

** *p* < 01.

Parentheses indicate 95% confidence intervals

1 PrEP Stigma estimates reflect a 50-point increase on the scale, as this was the approximate interquartile range of participant responses Estimates where *p* < 0.05 are bolded. Estimates where 0.05< *p* < 0.10 are italicized

**Table 3:** Themes related to PrEP stigma in interviews with black sexual minority men (*n*=23)

PrEP-specific sexual stigma (91.3%)	Anticipated sexual stigma (87.0%), Experienced sexual stigma (52.2%), Internalized sexual stigma (52.2%)

Intersections between PrEP and HIV stigma (34.8%)	General HIV stigma related to PrEP (34.8%), Intracommunity concerns about presumptions for having HIV (26.1%), External assumptions about HIV due to race and sexuality (21.7%)
PrEP misinformation and disinformation (34.8%)	General misinformation/disinformation (34.8%), Overestimating PrEP harms/difficulties (17.4%), Underestimating PrEP benefits (13.0%),

Parentheses after each theme contain the percentage of participants interviews where that theme was observed

## Appendix 1

Characteristics among Total Quantitative Sample (*n*=151) and Qualitative Subsample (*n*=23)

**Table:**

	Quantitative Sample (%)	Qualitative Sub-sample (%)
**Age group**
18–24	12.7	21.7
25–34	48.1	52.2
35–49	39.2	26.1
**Highest education level**
High school or less	8.9	8.7
Some college	22.8	26.1
College (undergraduate degree)	32.9	34.8
College (graduate degree)	35.4	30.4
**Region**
Northeast	59.5	60.9
West	5.1	4.3
Midwest	3.8	0.0
South	31.7	34.8
**Sexual identity**
Bisexual	20.3	26.1
Gay	63.3	43.5
Heterosexual	3.8	8.7
Blaqueer/SGL/Queer	12.7	21.7
**Relationship status**
Single	44.3	34.8
Dating	17.7	17.4
Partnered—monogamous	31.7	34.8
Partnered—non-monogamous	6.3	13.0
**Current health insurance**
Yes	84.8	78.3
No	15.2	21.7
**Sexual partner concurrence**
Yes	56.9	47.8
No	43.1	52.2
**Depression above median**
Yes	56.3	52.2
No	43.7	47.8
**Current PrEP use**
Yes	28.5	34.8
No	71.5	65.2

All proportion differences between total quantitative sample and qualitative subsample were <10%, except for age 35 or older (13.1% proportion difference) and gay sexual identity (19.8% proportion difference)
